# Chondromyxoid fibroma-like osteosarcoma: a case series and literature review

**DOI:** 10.1186/s12891-020-3063-5

**Published:** 2020-01-29

**Authors:** Jingyu Zhong, Liping Si, Jia Geng, Yue Xing, Yangfan Hu, Qiong Jiao, Huizhen Zhang, Weiwu Yao

**Affiliations:** 1 Department of Imaging, Tongren Hospital, Shanghai Jiao Tong University School of Medicine, 1111 Xian Xia Road, Shanghai, 200050 China; 2 Department of Radiology, Shanghai Jiao Tong University Affiliated Sixth People’s Hospital, 600 Yi Shan Road, Shanghai, 200233 China; 3 Department of Pathology, Shanghai Jiao Tong University Affiliated Sixth People’s Hospital, 600 Yi Shan Road, Shanghai, 200233 China

**Keywords:** Osteosarcoma, Low-grade central osteosarcoma, Chondromyxoid fibroma-like osteosarcoma, Chondromyxoid fibroma

## Abstract

**Background:**

Chondromyxoid fibroma-like osteosarcoma (CMF-OS) is an exceedingly rare subtype of low-grade central osteosarcoma (LGCO), accounting for up to 10% of cases and making it difficult to diagnose. CMF-OS is frequently misdiagnosed on a radiological examination and biopsy, even after the initial operation. Its treatment is a controversial issue due to its low-grade classification and actual high-grade behavior.

**Case presentation:**

We retrospectively reviewed the medical charts of more than 2000 osteosarcoma patients between 2008 and 2019; 11 patients with CMF-OS were identified, of which six patients were treated by our institution with complete clinical characteristics, including treatment and prognosis, radiological and pathological features were reviewed. Three males and three females with a median age of 46 (range 22–56) years were pathologically proven to have CMF-OS. The radiological presentation of CMF-OS is variable, thus radiological misdiagnoses are common. However, one must not ignore a malignant radiologic appearance. The most distinctive pathological feature conferring the diagnosis of CMF-OS is the presence of osteoid production directly by the tumor cells under a chondromyxoid fibroma (CMF)-like background. Differential diagnoses based on comprehensive data from CMF, LGCO, chondrosarcoma (CHS), conventional osteosarcoma (COS), etc., are needed. All patients were treated with an operation and chemotherapy, and one patient received additional radiotherapy. Nevertheless, recurrence and metastasis are common in CMF-OS patients. Relatively invasive biological behavior of CMF-OS is against the low-grade classification of this disease.

**Conclusions:**

It is important to recognize CMF-OS and distinguish it from CMF, CHS, COS and other LGCOs. CMF-OS has a relatively poor prognosis despite its low-grade classification.

## Background

Conventional osteosarcoma (COS) is the most common primary high-grade sarcoma of the skeleton in which neoplastic cells produce bone [1, 2]. It has a bimodal age distribution, with most cases developing between the ages of 10–14 years and a second smaller peak in older adults aged > 40 years. COS, commonly containing varying amounts of neoplastic cartilage and/or fibroblastic components, is subdivided into osteoblastic, chondroblastic and fibroblastic variants according to specific histological features based on the predominant matrix.

There are also several rare histological subtypes of osteosarcoma, including the giant cell rich variant, the osteoblastoma-like variant and the epithelioid variant, etc., in which four variants were defined by Mirra et al. [3] as low-grade osteosarcoma. This group of osteosarcoma displaying low-grade histological and biological behavior is comprised of parosteal osteosarcoma-like or fibrous dysplasia-like, non-ossifying fibroma-like, osteoblastoma-like and chondromyxoid fibroma-like variants, of which the metastatic rate is up to 10%. Among these subtypes, chondromyxoid fibroma-like osteosarcoma (CMF-OS) is the rarest. The first case was firstly reported in 1989 [3] as a subtype of low-grade osteosarcoma. Ten cases were reported in the literature to date [3–10].

CMF-OS was defined as a subtype of COS in the *World Health Organization classification of tumors, Pathology and genetics of tumors of soft tissue and bone* in 2002 [1], but was not listed in the 4th edition in 2013 [2]. Due to its rarity and overlap of characteristics with other lesions, CMF-OS can be easily misdiagnosed. However, it is important to distinguish CMF-OS from CMF and other lesions because of their different biological behaviors and associated therapeutic implications [4].

Herein, we report a case series of six CMF-OS patients with detailed clinicopathological and radiological features and review the literature to increase our recognition of this rare tumor. The differential diagnosis, treatment and prognosis of this tumor are also discussed.

## Case presentation

We retrospectively investigated the electronic charts of patients with a histological diagnosis of osteosarcoma admitted to Shanghai Jiao Tong University Affiliated Sixth People’s Hospital (Shanghai, China) between January 2008 and May 2019. From among more than 2000 osteosarcoma patients, only 11 were proven to have CMF-OS, of which five were consulting cases and thus additional clinical details and follow-up data are not available. Accordingly, six patients were recruited for the final analysis. The pathological diagnoses were carefully reviewed by two pathologists (QJ and HZ) who specialized in musculoskeletal pathology for 10 and 30 years based on haematoxylin-eosin staining and additional immunohistochemistry (IHC) staining. Imaging findings were reviewed by a radiology resident (JZ) and a radiologist (WY) with 30 years of image diagnostic experience of musculoskeletal diseases based on all available data. The relevant clinical features and related treatments were retrospectively reviewed, and follow-up data were collected from electronic charts, hard copy medical records and telephone calls. The review of the literature available via the PubMed, Embase and MEDLINE in English and China National Knowledge Infrastructure and Wanfang Data in Chinese revealed ten case reports with pathologically confirmed CMF-OS and had sufficient data to extract [3–10]. The institutional review board of our institution approved this retrospective study. Written consents were obtained from all patients included in this study.

The patients’ baseline characteristics are summarized in Table 1. Additionally, radiographic and pathological images supporting the conclusions of this study are available from the corresponding author on reasonable request.

**Table 1:** Characteristics and follow-up of 6 patients with CMF-OS

No.	Age	Sex	Site	Symptom (duration)	Treatment (times)	Follow-up (after surgery)
1	21	F	proximal left femur	weakness, pain, 1 mo	C*4 + S*1 + C*1	metastasis to the lung, 6 mo
2	37	F	left 2nd rib	mass, 3 mo	S*1 + C*12 + R*1	DFS, 20 mo
3	56	F	3rd toe	swelling, pain, 12 mo	S*1 + C*12	DFS, 39 mo
4	51	M	nasal cavity	mass, 0.5 mo	S*2 + C*7	recurrence, 13 mo; AWD, another 8 mo
5	27	M	right mandible	mass, pain, 2 mo	S*2 + C*1	DFS, 4 mo
6	50	M	left maxilla	mass, 2 mo	S*2 + C*8	metastasis to the lung, 2 mo; AWD, another 7 mo

*F* Female, *M* Male, *S* Surgery, *C* Chemotherapy, *R* Radiotherapy, *AWD* Alive with disease, *DFS* Disease-free survival, *mo* Months

## Case 1

**Fig. 1:**
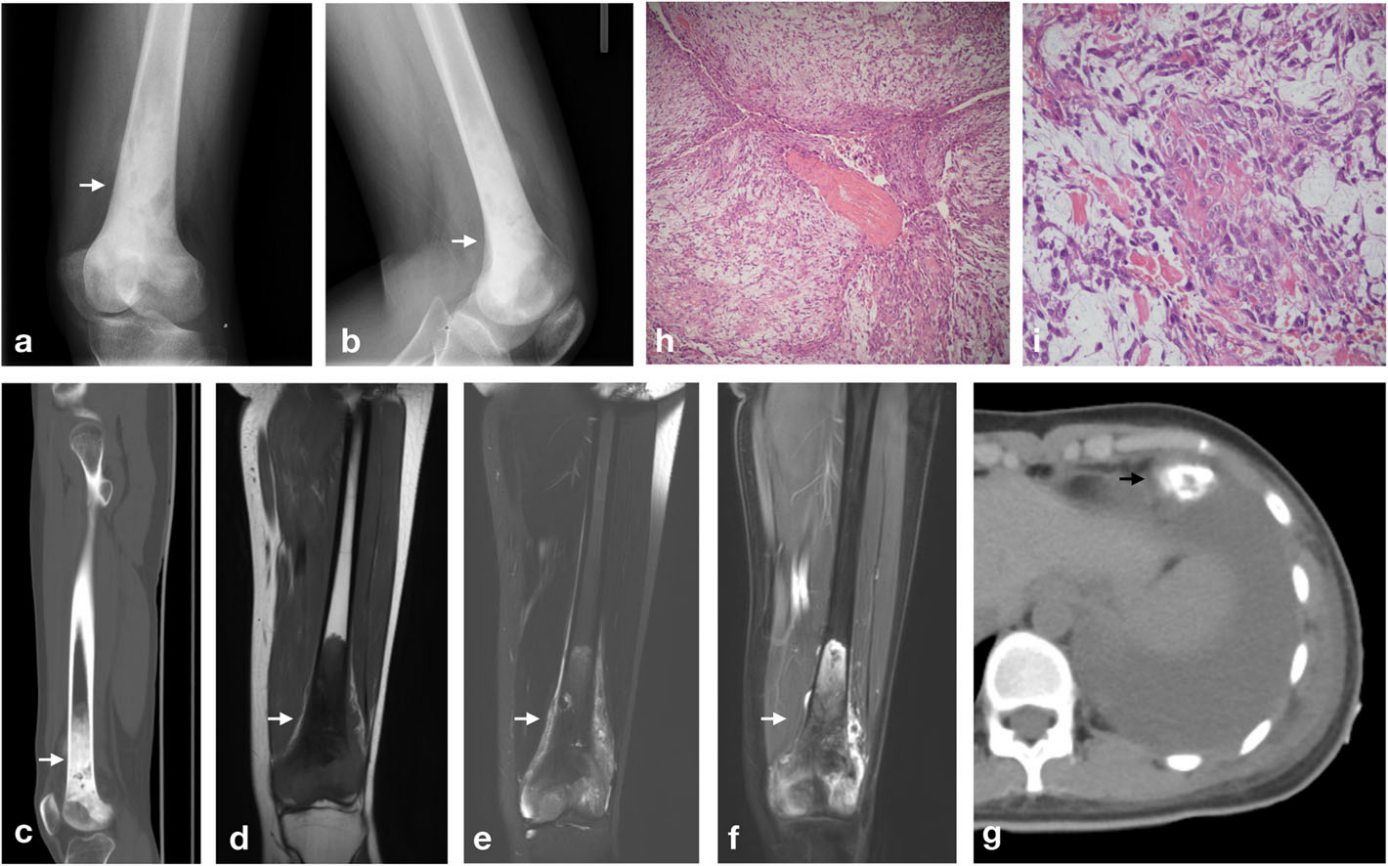
Radiological and pathological findings of case 1 (**a**), (**b**) CR showed an osteogenic lesion similar to that on sagittal CT (**c**) on bone windows through the femur. (**d**) and (**e**) MRI demonstrated an isointense lesion to muscle tissue with a low signal intensity centre on T1-weighted images and a heterogeneous isointense to hyperintense lesion on T2-weighted images. (**f**) After contrast administration, the lesion showed heterogeneous peripheral enhancement with a foci of low signal intensity centrally on post-contrast images. (**g**) Six months after surgery, nodules with calcification and ossification accompanied by pleural effusion were found in her lung. (**h**) and (**i**) Haematoxylin-eosin staining of resected specimens showed loose aggregates of cells in a highly myxoid stroma background and segregated into lobules by the fibrovascular septa; however, under high-power microscopy, the presence of osteoid production directly by the tumour cells and nuclear abnormalities were found

A 21-year-old female presented with a one-month history of pain and weakness of her left knee. A physical examination (PE) showed obvious swelling and limitations in mobility. Significant lab test results included an elevated alkaline phosphatase (ALP) level of 240 U/L. Computed radiography (CR) showed an osteogenic lesion in the distal femur and an associated soft tissue mass. Computed tomography (CT) findings were similar to those of CR. Magnetic resonance imaging (MRI) demonstrated abnormal signals in the distal femur on T1-weighted and T2-weighted images (Fig. 1a-g). The radiological diagnosis was osteosarcoma, which agreed with the biopsy results. She received four cycles of neoadjuvant chemotherapy before surgery, and the necrosis rate of the tumor was only 10%, indicating a poor prognosis. She was confirmed to have CMF-OS (Fig. 1h) and received one cycle of adjuvant chemotherapy after surgery. Six months later, several nodules accompanied by pleural effusion were found in her lung and confirmed as metastatic tumors. The patient received no further treatment and refused further follow-up.

## Case 2

**Fig. 2:**
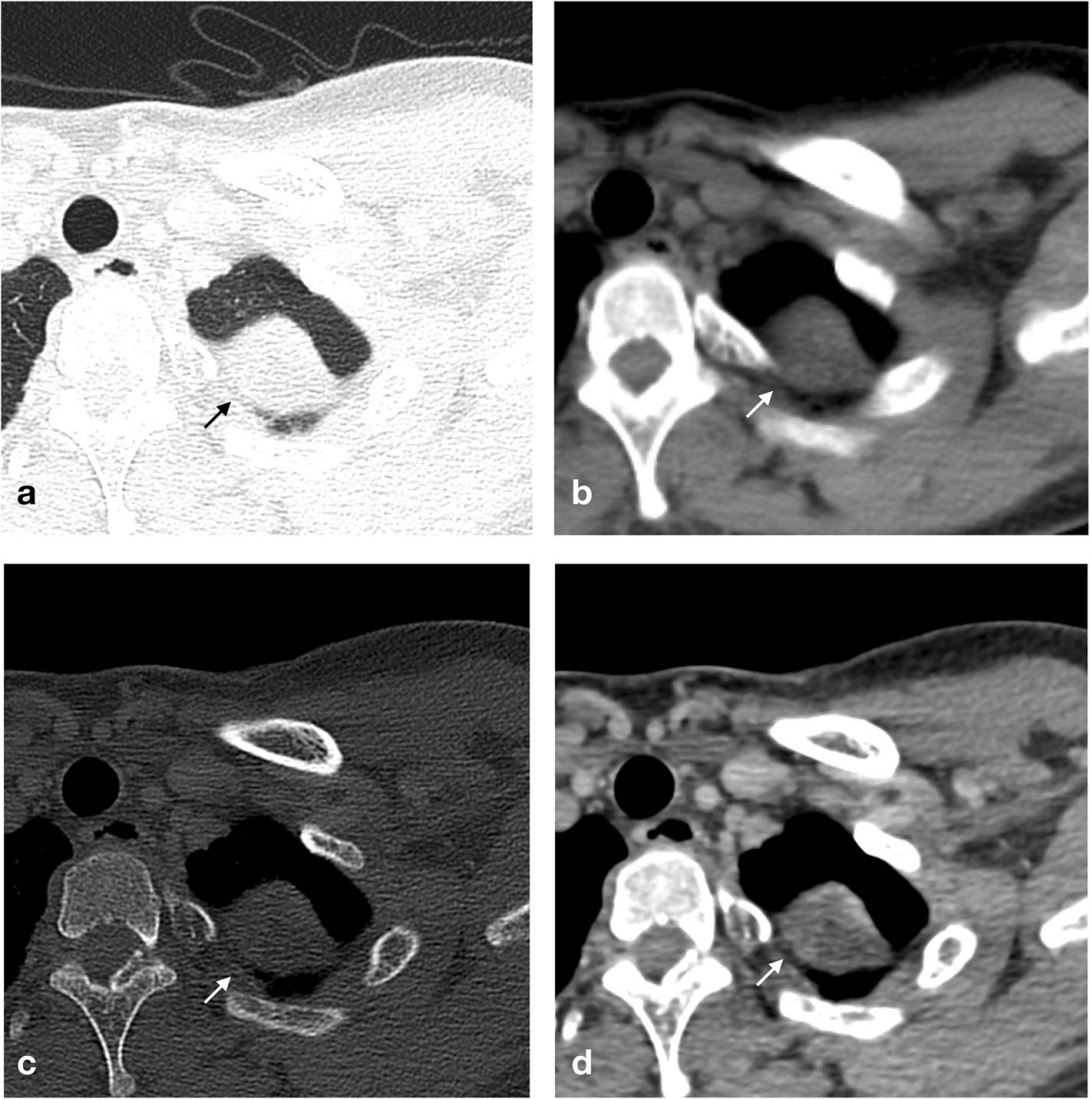
Radiological and pathological findings of case 2 (**a-c**) Axial CT on the lung, bone and soft tissue windows showed an expansive lesion with destruction and a pathological fracture, as well as an associated soft tissue mass. (**d**) After contrast administration, enhancement of the mass was demonstrated

A 37-year-old previously healthy female discovered a mass of her left second rib with no symptoms on CR at an annual medical test. A further CT scan showed an expansive lesion. MRI showed a lesion measuring 3.3 × 2.7 cm in the left apex of the lung. The radiological diagnosis was initially fibrous dysplasia. Positron emission tomography/computed tomography (PET/CT) showed an expansive lesion with sclerosing and cystic changes that was considered fibrous dysplasia or a chondrogenic tumor. CT scan after enhancement demonstrated bone destruction and a soft tissue mass, and the patient was diagnosed with a fibrogenic or chondrogenic tumor (Fig. 2a-c). Three months after discovery, her tumor was removed with surgery and pathologically confirmed as CMF-OS (Fig. 2d). She was then treated with 12 cycles of adjuvant chemotherapy and started to receive radiotherapy. The patient survived more than 19 months after surgery and is still receiving the appropriate treatment.

## Case 3

A 56-year-old female first presented with swelling and pain in her right foot for more than a year. PE discovered a mass of approximately 1.0 × 1.0 cm with tenderness and increased skin temperature. CT showed destruction and sclerosing of the basal part of the right third metatarsal bone and lateral cuneiform bone with soft tissue swelling. MRI was performed to delineate the soft tissue and revealed a mass with abnormal signals and destruction of surrounding bones (Additional file 1: Figure S1). The radiological diagnosis was a synovial origin tumor (giant cell tumor of tendinous sheath), and the biopsy result was a low-grade malignant mesenchymal tumor (mixed tumor), while the final diagnosis after surgery was CMF-OS. She was given 12 cycles of adjuvant chemotherapy after surgery. The patient made an uneventful recovery and remained well during a 38-month follow-up.

## Case 4

A 51-year-old male presented with nasal obstruction and nasal discharge with a history of a mass in his nasal cavity that was surgically removed 13 months prior. He was a heavy smoker and received no adjuvant chemotherapy. The former mass was pathologically identified as CMF-OS. When he came to us, the CT and MRI scan showed a nasopharyngeal soft tissue mass of approximately 5.6 × 4.5 × 4.5 mm (Additional file 2: Figure S2). Taking his medical history into consideration, recurrence was confirmed. A biopsy of the second mass was performed, which revealed CMF-OS with dedifferentiated components of high-grade fusocellular sarcoma. He could not be treated with a re-operation and consequently received seven cycles of chemotherapy instead. He survived with a tumor after recurrence for another 7 months.

## Case 5

A 27-year-old male with a mass on his right mandible was clinically misdiagnosed as having pericoronitis in a local hospital and initially treated with anti-inflammation therapy. Due to no obvious improvement, an operation was performed to remove the mass and adjacent teeth, and the mass was histologically confirmed as CMF-OS. Therefore, a re-operation was performed 1 month later to reach the negative margin. When he came to us for further treatment, radiological images showed no recurrence (Additional file 3: Figure S3). Three months after the operation, two cycles of adjuvant chemotherapy were already completed, and the next cycle was continued. The patient remained well on follow-up.

## Case 6

A 50-year-old male with a 2-month history of a left maxilla mass of approximately 3.0 × 3.5 cm received an operation to resect the mass, and then a re-operation was performed to remove the residual disease, which was pathologically confirmed as CMF-OS. Two months later, the patient presented to us for further treatment. A CT scan showed no recurrence, but several scattered nodules were discovered in his lung, which were considered metastases (Additional file 4: Figure S4). Eight cycles of chemotherapy were completed, and the disease showed no progression, but the patient refused follow-up after the latest cycle of chemotherapy.

## Discussion and conclusion

CMF-OS is a rare type of osteosarcoma, classified as a subtype of COS [1, 2], and also considered as a subtype of LGCO [3]. There is no specific clinical or radiological features demonstrated before. Histologically, it is characterized by loose aggregates of cells supported in a highly myxoid stroma and segregated into lobules by fibrovascular septa, with osteoid production by the tumor cells suggesting osteosarcoma [4]. However, all of the cases were misdiagnosed radiologically, and none of the pathological diagnoses at biopsy were matched to the final diagnosis at surgical resection, highlighting the difficulty of this diagnosis. The treatment varies, including surgical resection, chemotherapy and radiotherapy. We described six cases of CMF-OS in details, to the best of our knowledge, that is the studies with the largest number of cases, and summarizes 16 cases of CMF-OS [3–10] in Table 2.

**Table 2:** Summary of 16 cases of CMF-OS, including the present cases

No.	Age	Sex	Site	Radiology	Biopsy	Follow-up	Reference
1	34 y	F	5th finger	unknown	CMF	recurrence, 4 y	Mirra, 1989 [3]
2	unknown	unknown	unknown	unknown	CMF	recurrence, 12 y	Mirra, 1989 [3]
3	39 y	F	maxilla	soft tissue mass	myxoma	recurrence, 4 y	Chow, 1996 [4]
4	39 y	M	left iliac crest	osteolytic lesion; soft tissue mass with calcification	CHS	metastasis to the lung and liver, 6 y	Chow, 1996 [4]
5	25 y	F	right mandible	diffuse mixed-density lesion	OS	DFS, unknown	Sinha, 2010 [5]
6	45 y	M	left maxilla	poorly defined areas of sclerosis with few lytic areas	CMF	recurrence and metastasis to the brain, 5 mo	Prasad, 2015 [6]
7	13 mo	F	right maxillary sinus and nasal cavity	soft tissue mass with mild contrast enhancement and internal calcifications	CMF	AWD, 2 y	Lee, 2015 [7]
8	13 y	M	proximal right tibia metadiaphysis	large lytic, septate lesion with cortical destruction; soft tissue mass	OS	DFS, 6 mo	Stark, 2017 [8]
9	9 y	F	left femur	osteolytic, osteogenic lesion; soft tissue mass	OS	DFS, 6 mo	Liu, 2007 [9]
10	39 y	M	left femur	soft tissue mass	CHS	DFS 6 mo	Ma, 2016 [10]
11	21 y	F	proximal left femur	osteogenic lesion	OS	metastasis to the lung, 7 mo	Present case
12	37 y	F	left 2nd rib	expansive lesions	CMF	DFS, 20 mo	Present case
13	56 y	F	3rd toe	destruction, soft tissue mass	mixed tumour	DFS, 39 mo	Present case
14	51 y	M	nasal cavity	soft tissue mass	CMF	recurrence, 13 mo; AWD, another 8 mo	Present case
15	27 y	M	right mandible	soft tissue mass	OS	DFS, 4 mo	Present case
16	50 y	M	left maxilla	soft tissue mass	CMF	metastasis to the lung, 2 mo; AWD, another 7 mo	Present case

*F* Female, *M* Male, *CMF-OS* Chondromyxoid fibroma-like osteosarcoma, *CMF* Chondromyxoid fibroma, *CHS* Chondrosarcoma, *OS* Osteosarcoma, *AWD* Alive with disease, *DFS* Disease-free survival, *y* Years, *mo* Months

## Clinical features

Fifteen patients (eight females and seven males) with detailed information were reviewed. CMF-OS involved almost equally in both gender, in contrast to what is typically observed in COS; however, this could be the result of the deficient number of patients. Of these patients, 12 were adults, and three were pediatrics, with ages ranging from 13 months to 56 years (median 37 years), distinct from that observed in COS. In our cases, there are three patient over 50 years suffered from CMF-OS, demonstrated that CMF-OS can occur in middle-aged adults.

As a subtype of osteosarcoma, CMF-OS is less commonly found in the long bones of the extremities. In the present case series, only three tumors were located surrounding the knee, the typical site of COS, including the distal femur and proximal tibia. The rest were found in the proximal femur. Accordingly, CMF-OS is more commonly found in craniofacial areas, where COS is uncommonly involved. In fact, seven of fifteen tumors in our summary were located in the craniofacial area. Occasionally, CMF-OS can be found in other sites, including short and flat bones, but it is rare for osteosarcoma to be found in such sites, making it easy to mistake for other lesions. Of the ten cases described previously, only one tumor reported by Chow et al. [4] was found in the left iliac crest, a flat bone. We reported the first CMF-OS occurring in the rib and the second occurring in a flat bone. Remarkably, it was the first case of no symptoms and was discovered during an occasional medical check-up.

Most patients presented with a symptomatic mass, swelling or pain, while one patient discovered the asymptomatic mass at an annual medical test. Tumors in the craniofacial area can initiate oral or nasal problems. Although ALP and lactate dehydrogenase (LDH) levels may increase in patients with osteosarcoma and have been proven to be prognostic factors for osteosarcoma [11, 12], the laboratory test showed no specified results; only one patient in our case series showed an elevated ALP level, and no patient had an abnormal LDH level. There is no abnormal laboratory results reported in previous studies, indicating limited value of ALP or LDH level in diagnosis or prognosis of CMF-OS.

## Radiological characteristics

We observed several distinct radiological features that enable recognition in an appropriate clinical setting. As a tumor comprised of several components, the radiological presentation of CMF-OS is variable. CMF-OS can present as an osteolytic, osteogenic or expansive lesion with or without calcification with soft tissue masses. Evidence of well, moderately or poorly defined delineation and the appearance of a thin, broken or destructive cortex are also found. A lesion presenting as a large, destructive, poorly defined, mixed lytic and blastic mass that transgresses the cortex and forms a very large soft tissue mass [1], similar to the typical appearance of COS, is not difficult to diagnose as a malignant diseases. One of our patients and two of previous cases [8, 9] presented with an obvious osteogenic lesion surrounding the knee were diagnosed as osteosarcoma.

Unfortunately, most of the tumors showed with no distinguishable characteristics, making it difficult to identify their histological origin. However, their relatively invasive appearance indicated to be potentially malignant. CMF-OS involving the craniofacial area (as case 5) is common and confusing. It sometimes could be misdiagnosed as CMF, which is considered as an intermediate tumor with a much better prognosis. The majority of CMFs show radiological features of benign bony lesions consisting of internally lobulated to lobulated translucent osteolytic defects with scalloped and sclerotic margins [13]. In contrast, CMF-OSs may display radiographic features suggesting malignancy. Other cases showed a similar appearance but lacked characteristics in atypical sites, leading to a misdiagnosis of chondrogenic tumors, fibrogenic tumors, synovial origin tumors and even infection. Under these conditions, careful and comprehensive studies of clinical, radiological and pathological features are necessary.

## Pathological findings

Several distinct pathological features may enable the recognition of such tumors in the appropriate clinical setting. Grossly, a glistening and mucoid cut surface of the tumor reflects a highly myoxid stroma. Microscopically, it shows a CMF-like appearance, including loose aggregates of cells in a highly myxoid stroma background and segregated into lobules by the fibrovascular septa. Furthermore, the most distinctive feature conferring the diagnosis of osteosarcoma is the presence of osteoid production directly by the tumor cells [4]. Moreover, nuclear abnormalities and an elevated Ki-67 index [6] are also helpful. However, a pathological diagnosis is also of great difficulty based on limited tissue obtained from a biopsy. To avoid this situation, one must not ignore a malignant radiologic appearance. In addition, MDM2 and CDK4 were reported to be valuable in differentiating LGCO from other primary fibroosseous lesions of the bone [14].

Differential diagnoses of CMF-OS include CMF, chondrosarcoma (CHS), other LGCOs and COS, as shown in Table 3 [2, 14–18]. The most distinctive feature in differentiating between CMF and CMF-OS, that should be carefully searched, is neoplastic bones, especially in lesions with malignant radiological features. IHC staining for Vimentin-positive and S100-negative cells may be helpful [4]. CHS is another malignant tumor that may be confused with CMF-OS; however, mineralization on a CT scan and ring-and-arc enhancement on an MRI may suggest its chondrogenic origin [15, 16]. Because of the relatively invasive biological behavior of CMF-OS, a differential diagnosis from other LGCOs and COS has clinical significance and is associated with appropriate therapeutic implications [17].

**Table 3:** Differential diagnostic features

Characteristic	CMF-OS	CMF	LGCO	CHS	COS
Age (years)	All ages	All ages, preferably < 30	30–40	> 40	10–14 and > 40
Site	Craniofacial part; femur	Proximal tibia, distal femur; ilium	Distal femur, proximal tibia	Bones of the pelvis; proximal femur, proximal humerus, distal femur; rib	Distal femur, proximal tibia, proximal humerus
Location in the long bone	Variable	Metaphysis; eccentric in long bones	Metaphysis, diametaphysis	Metaphysis or Diaphysis; Centric in long bones	Metaphysis
CR	poorly defined osteolytic, osteogenic or expansive lesion	Well-defined osteolytic lesion	Large, lytic coarsely trabeculated lesion with a focal aggressive feature	Osteolytic lesion with endosteal scalloping; cortical thickening	Large, poorly defined, mixed lytic and blastic mass
CT	Cortex destruction	Sclerotic margin	Cortex destruction; periosteal reaction	Mineralization; cortex erosion or destruction	Mineralization, ossification; cortex destruction; periosteal reaction
MRI	Soft tissue mass; Soft tissue and bone marrow oedema	Peripheral nodular enhancement; no bone marrow or soft tissue oedema	Soft tissue extension; difficult to distinguish from COS	Ring-and-arc enhancement; soft tissue oedema	Soft tissue mass; soft tissue and bone marrow oedema
Histology	Loose aggregates of cells supported in a highly myxoid stroma; Neoplastic bone	Lobular pattern with stellate or spindle-shaped cells in a myxoid background	Hypocellular to moderately cellular fibroblastic proliferation with variable amounts of osteoid production; neoplastic bone	Abundant blue-grey cartilage matrix production; atypical chondrocyte varying in size and shape with enlarged, hyperchromatic nuclei	Neoplastic cells with severe anaplasia and pleomorphism; neoplastic bone
Immunohistochemistry	Vimentin (+); S100 (−)	S100 (+); collagen II, SOX9 (+) in the centre of lobules	MDM2, CDK4 (+)	20% IDH1(+) with IDH R132H antibody	osteocalcin, osteonectin, S100, action, SMA, NSE, CD99 (+); Factor VIII, CD31, CD45 (−)
Genetic	None	Chromosome 6 aberration	Gain or amplification of MDM2 (12q13–15)	IDH1 mutation	Multiple numerical and structural chromosomal aberrations

*CMF-OS* Chondromyxoid fibroma-like osteosarcoma, *CMF* Chondromyxoid fibroma, *LGCO* Low-grade central osteosarcoma, *CHS* Chondrosarcoma, *COS* Conventional osteosarcoma

## Treatment and prognosis

Surgical resection is the primary treatment for CMF-OS. All of the cases we reviewed here were removed; nevertheless, only four patients received chemotherapy [4–6, 8] while one patient received radiotherapy [6] in previous study. In our institution, patients with CMF-OS are treated as COS, with multi-agent chemotherapy optimized by our oncology department [18]. Despite a similar routine, there was only a 10% tumor response to neoadjuvant chemotherapy in our cases, in contrast to the good response reported by Stark et al. [8].

Four of nine patients reported disease-free survival of only up to 6 months [5, 8–10], which is insufficient for illustrating its long-term prognosis. Recurrence or metastasis occurred in the remaining five patients 5 months to 12 years later [3, 4, 6], displaying its propensity to evolve into a high-grade osteosarcoma, One patient lived with residual tumor for 2 years [7]. Furthermore, three of our six patients were found to have recurrence or metastasis within 2 to 13 months after surgery, suggesting a high recurrence and metastasis rate, which were much higher than those reported by Mirra et al. [3] previously. Due to its relatively invasive biological behavior, limited response to treatment and the probability of recurrence and metastasis, it may be unsuitable for CMF-OS to be classified as LGCO.

CMF-OS is an extremely rare variant that is more commonly found in atypical sites of osteosarcoma with confusing clinical, radiographic and pathological features. Difficulties in diagnosis are the first hindrance to an appropriate treatment. Many patients did not reach to the correct diagnosis until the first surgery was completed and had to suffer the second one. The involvement of the craniofacial area may also play an important role in its poor prognosis. Almost half of tumors reported were found in the craniofacial area, where complete resection is difficult to accomplish. Indeed, osteosarcomas involving axial skeleton as an adverse factors for prognosis [19]. Last but the most important, CMF-OS was once considered a low-grade variant of osteosarcoma, which confused professionals and blocked the appropriate treatment and necessary long-term follow-up. In contrast, our summary showed that half of patients experienced recurrence or metastasis within 2 months to 12 years. To achieve an appropriate treatment and reasonable follow-up, further study of clarification of its classification based on its biological behavior and molecular background are necessary.

In conclusion, a case series of six patients with CMF-OS and related literature were reviewed in this study. The features that enable the diagnosis of CMF-OS were summarized, emphasizing a differential diagnosis between CMF, CHS, COS and other LGCOs. An appropriate treatment selection based on its biological behavior and probable cause of its relatively poor prognosis were discussed, highlighting the necessity for clarifying its classification.

## Supplementary information

**Additional file 1: Figure S1.** Figure-case-3 (a), (b) Axial CT on the bone and soft tissue windows through the right foot showed destruction and sclerosing of the bone and an associated soft tissue mass with rings and patched calcification. (c), (d) Coronal T1-weighted images demonstrated a homogeneous isointense to hypointense lesion to muscle tissue. (e), (f) Heterogeneous hyperintense lobular lesions were observed on coronal PD-weighted images.

**Additional file 2: Figure S2.** Figure-case-4 (a) Coronal T2-weighted images demonstrated a heterogeneous hyperintense lesion to the grey matter. (b), (c) Before and after contrast administration, the lesion was isointense to hypointense on axial T1-weighted images and then showed heterogeneous enhancement.

**Additional file 3: Figure S3.** Figure-case-5 (a), (b) Axial CT on the bone and soft tissue windows through the right mandible after resection showed no soft tissue mass.

**Additional file 4: Figure S4.** Figure-case-6 (a), (b) Axial T1-weighted and coronal PD-weighted images through the left maxilla demonstrated a soft tissue mass with a heterogeneous signal. (c) Several nodules found on axial CT were considered metastases.

## Data Availability

The datasets used and/or analyzed during the current study are available from the corresponding author on reasonable request.

## Abbreviations

ALP: Alkaline phosphatase
CHS: Chondrosarcoma
CMF: Chondromyxoid fibroma
CMF-OS: Chondromyxoid fibroma-like osteosarcoma
COS: Conventional osteosarcoma
CR: Computed radiography
CT: Computed tomograph
IHC: Immunohistochemistry
LDH: Lactate dehydrogenase
LGCO: Low-grade central osteosarcoma
MRI: Magnetic resonance imaging
PE: Physical examination
PET/CT: Positron emission tomography/computed tomography

## References

[1] Fletehe CDM, Unni KK, Mertens F (2002). Chapter 11 Osteogenic tumors. World Health Organization classification of tumors. Pathology and genetics of tumours af soft tissue and bone.

[2] Fletcher CDM, Bridge JA, Hogendoorn PCW, Mertens F (2013). Chapter 16 Osteogenic tumors. World Health Organization classification of tumors. WHO classification of tumours of soft tissue and bone.

[3] Mirra J, Picci P, Gold R (1989). Bone tumors: clinical, radiologic, and pathologic correlations.

[4] Chow LTC, Lin J, Yip KMH (1996). Chondromyxoid fibroma-like osteosarcoma: a distinct variant of low-grade osteosarcoma. Histopathology.

[5] Sinha R, Roy Chowdhury SK, Chattopadhyay PK, Rajkumar K (2010). Low-grade osteosarcoma of the mandible. J Maxillofac Oral Surg.

[6] Prasad K, Dexith J, Lalitha RM (2015). Maxillary osteosarcoma masquerading as chondromyxoid fibroma: report of a case. J Maxillofac Oral Surg.

[7] Lee HC, Luk WH, Lo A, Chow LTC (2015). A case of chondromyxoid fibroma-like osteosarcoma in a 13-month-old girl. SM J Radiol.

[8] Stark M, Heinrich SD, Sivashanmugam R, Mackey D, Wasilewska E, Craver R (2017). Pediatric chondromyxoid fibroma-like osteosarcoma. Fetal Pediatr Pathol.

[9] Liu HH, Zhang JX, Du P (2007). Chondromyxoid fibroma-like osteosarcoma: a case report and review of literature. J Clin Exp Pathol.

[10] Ma YT, Shen DH (2016). Osteosareoma with chondromyxoid fibroma-like morphological characteristics: clinical and pathological analyses. J Diag Pathl.

[11] Bacci Gaetano, Longhi Alessandra, Ferrari Stefano, Briccoli Antonio, Donati Davide, De Paolis Massimiliano, Versari Michela (2004). Prognostic Significance of Serum Lactate Dehydrogenase in Osteosarcoma of the Extremity: Experience at Rizzoli on 1421 Patients Treated over the Last 30 Years. Tumori Journal.

[12] Bacci G, Longhi A, Versari M, Mercuri M, Briccoli A, Picci P (2006). Prognostic factors for osteosarcoma of the extremity treated with neoadjuvant chemotherapy: 15-year experience in 789 patients treated at a single institution. Cancer.

[13] Cappelle S, Pans S, Sciot R (2016). Imaging features of chondromyxoid fibroma: report of 15 cases and literature review. Br J Radiol.

[14] Dujardin F, Binh MBN, Bouvier C (2011). MDM2 and CDK4 immunohistochemistry is a valuable tool in the differential diagnosis of low-grade osteosarcomas and other primary fibro-osseous lesions of the bone. Mod Pathol.

[15] Douis H, Saifuddin A (2012). The imaging of cartilaginous bone tumours. I. Benign lesions. Skeletal Radiol.

[16] Vahlensieck M, Reiser M (2015). Chapter 12 Bone and soft tissue tumors. MRI of musculoskeletal system.

[17] Klein MJ, Siegal GP (2006). Osteosarcoma: anatomic and histologic variants. Am J Clin Pathol.

[18] Lin F, Wang Q, Yu W (2011). Clinical analysis of Chinese limb osteosarcoma patients treated by two combinations of methotrexate, cisplatin, doxorubicin and ifosfamide. Asia Pac J Clin Oncol.

[19] Smeland S, Bielack SS, Whelan J (2011). Survival and prognosis with osteosarcoma: outcomes in more than 2000 patients in the EURAMOS-1 (European and American Osteosarcoma Study) cohort. Eur J Cancer.

