# Supplementary figures and images for: Chondromyxoid fibroma-like osteosarcoma: a case series and literature review
Source: BMC Musculoskelet Disord. 2020 Jan 29;21:53. doi: 10.1186/s12891-020-3063-5 (PMC6990471; doi:10.1186/s12891-020-3063-5)

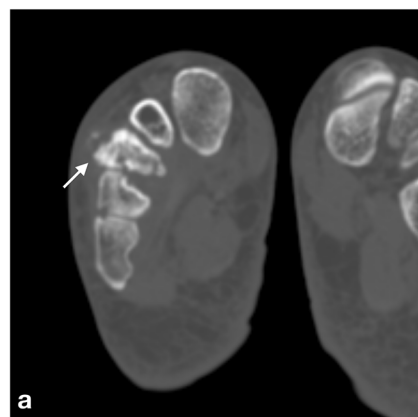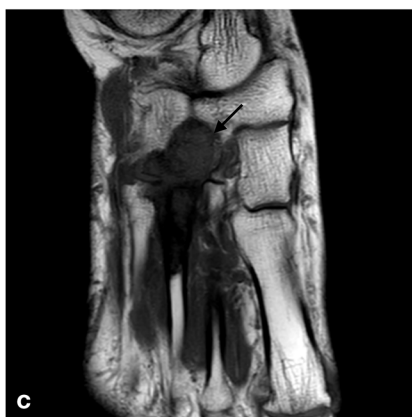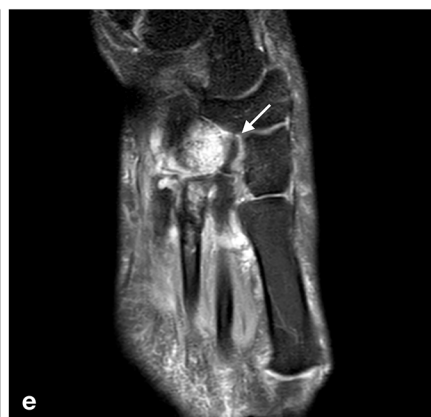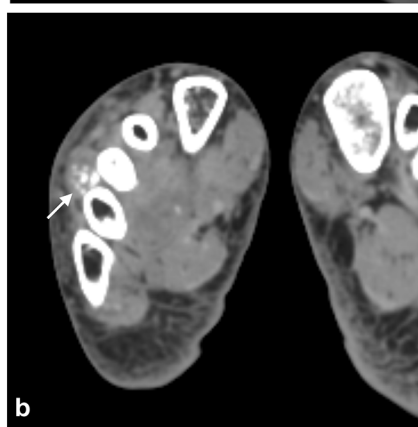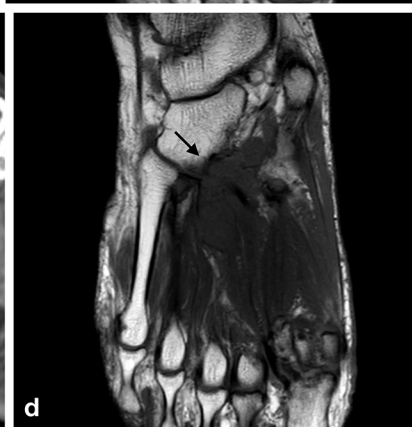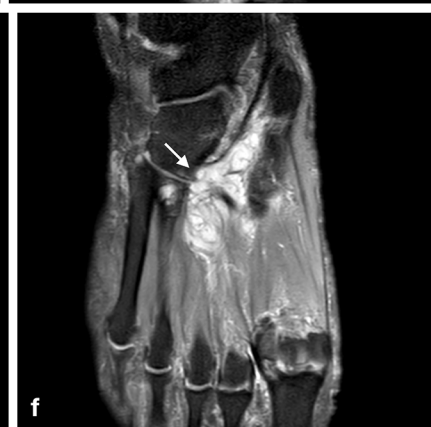

Supplement: Supplementary file 1 — Additional file 1: Figure S1. Figure-case-3 (a), (b) Axial CT on the bone and soft tissue windows through the right foot showed destruction and sclerosing of the bone and an associated soft tissue mass with rings and patched calcification. (c), (d) Coronal T1-weighted images demonstrated a homogeneous isointense to hypointense lesion to muscle tissue. (e), (f) Heterogeneous hyperintense lobular lesions were observed on coronal PD-weighted images. [file 12891_2020_3063_MOESM1_ESM.pdf]

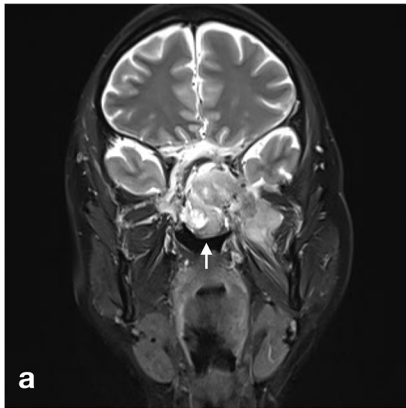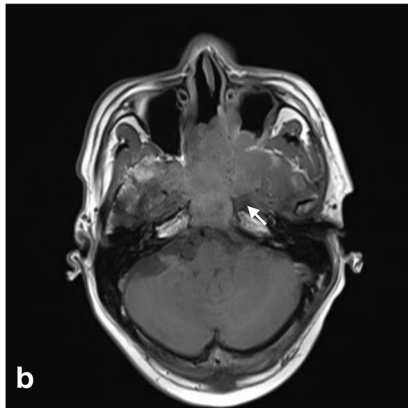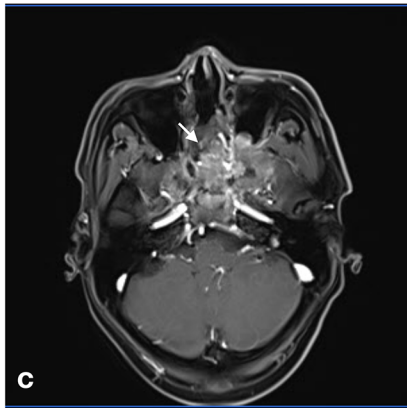

Supplement: Supplementary file 2 — Additional file 2: Figure S2. Figure-case-4 (a) Coronal T2-weighted images demonstrated a heterogeneous hyperintense lesion to the grey matter. (b), (c) Before and after contrast administration, the lesion was isointense to hypointense on axial T1-weighted images and then showed heterogeneous enhancement. [file 12891_2020_3063_MOESM2_ESM.pdf]

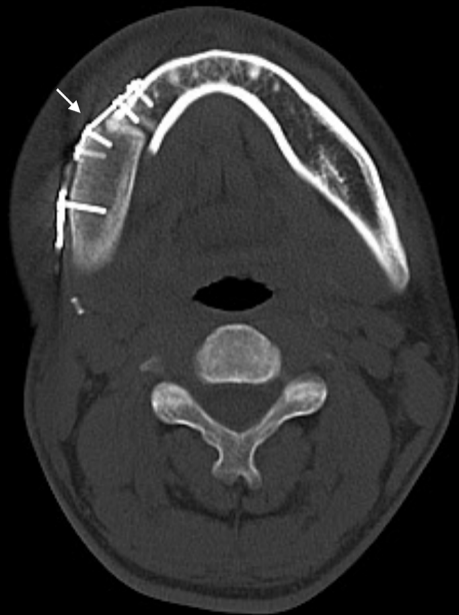

**a**

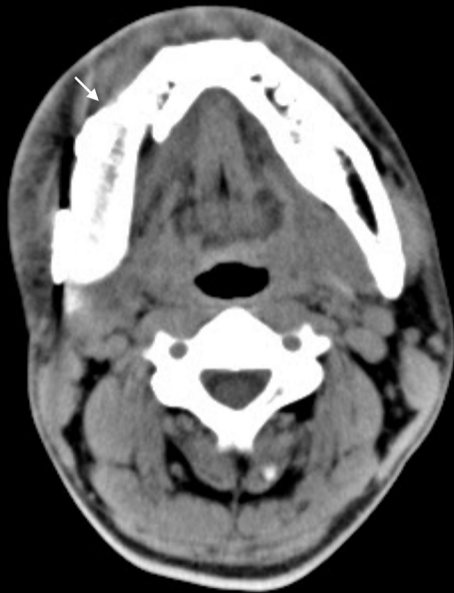

**b**

Supplement: Supplementary file 3 — Additional file 3: Figure S3. Figure-case-5 (a), (b) Axial CT on the bone and soft tissue windows through the right mandible after resection showed no soft tissue mass. [file 12891_2020_3063_MOESM3_ESM.pdf]

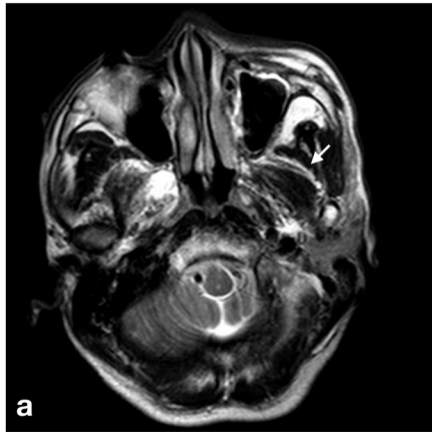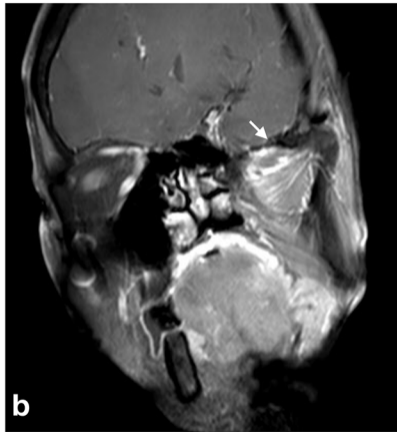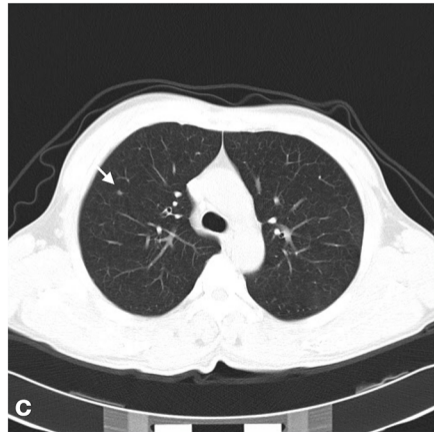

Supplement: Supplementary file 4 — Additional file 4: Figure S4. Figure-case-6 (a), (b) Axial T1-weighted and coronal PD-weighted images through the left maxilla demonstrated a soft tissue mass with a heterogeneous signal. (c) Several nodules found on axial CT were considered metastases. [file 12891_2020_3063_MOESM4_ESM.pdf]
